# Oropharyngeal Dysphagia in Acute Cervical Spinal Cord Injury: A Literature Review

**DOI:** 10.1007/s00455-022-10535-0

**Published:** 2022-11-14

**Authors:** Jackie McRae, Sarah Morgan, Emma Wallace, Anna Miles

**Affiliations:** 1 Centre for Allied Health, St George’s University of London, Cranmer Terrace, London, SW17 0RE UK; 2 Speech & Language Therapy Department, North West Regional Spinal Injuries Centre, Southport & Ormskirk NHS Trust, Southport, UK; 3 Discipline of Speech Pathology, Faculty of Medicine and Health, The University of Sydney, Sydney, Australia; 4 Speech Science, The University of Auckland, Auckland, New Zealand

**Keywords:** Dysphagia, Deglutition, Cervical spinal cord injury, Speech and language therapy, Risk factors

## Abstract

Dysphagia (swallowing impairment) is a frequent complication of cervical spinal cord injury (cSCI). Recently published national guidance in the UK on rehabilitation after traumatic injury confirmed that people with cSCI are at risk for dysphagia and require early evaluation while remaining nil by mouth [National Institute for Health and Care Excellence. Rehabilitation after traumatic injury (NG211), 2022, https://www.nice.org.uk/guidance/ng21]. While the pathogenesis and pathophysiology of dysphagia in cSCI remains unclear, numerous risk factors have been identified in the literature. This review aims to summarize the literature on the risk factors, presentation, assessment, and management of dysphagia in patients with cSCI. A bespoke approach to dysphagia management, that accounts for the multiple system impairment in cSCI, is presented; the overarching aim of which is to support effective management of dysphagia in patients with cSCI to prevent adverse clinical consequences.

**Supplementary Information:**

The online version contains supplementary material available at 10.1007/s00455-022-10535-0.

## Introduction

Rates of spinal cord injury (SCI) vary around the world with The World Health Organization (WHO) estimating between 250,000 and 500,000 injuries a year [2]. Annual incidence of SCI in the USA is reported to be 12,500 [3] while in the UK is estimated around 2500 [4]. Injuries are broadly categorized as being of traumatic or non-traumatic etiology [5]. Traumatic injuries commonly occur following either low-impact injury such as falls, or high impact, such as road traffic accident or sports injury. Non-trauma causes include spinal bleeds, tumors, or abscesses. A high survival rate contributes to a high worldwide burden of care and need for access to expert clinical services and effective interventions [6]. The mechanisms for damage to the cord can be primary, taking place at the time of injury or secondary, occurring in the aftermath of the injury due to physiological processes or clinical management. Injuries are described according to the neurological level of injury and severity. A classification system developed by the American Spinal Injury Association [7] is used following assessment by a trained healthcare professional within 72 h of injury. cSCI injuries are the most common, with impacts on upper limb functions, respiratory function, and autonomic functions [8].

Dysphagia is a common complication following SCI, with a greater probability following cSCI than damage to other levels of the spinal cord [9–11]. Dysphagia has life-threatening consequences, including aspiration pneumonia, the leading cause of death following cSCI [9, 12, 13]. The prevalence rates of dysphagia in cSCI range from 16 to 60% [11, 14, 15]. This large discrepancy is likely due to differences in etiologies, surgical management, dysphagia definitions, methods of diagnosis (e.g., dysphagia screening tool versus instrumental assessment), and variable timepoints in which dysphagia is evaluated and identified post injury.

Despite its high prevalence, the precise underlying mechanisms and pathophysiology of dysphagia in cSCI are poorly understood [9]. Dysphagia in cSCI may be (1) directly related to the neural injury itself, (2) the result of subsequent interventions (i.e., surgeries and spinal hardware), or (3) related to post-surgical complications of injury management (i.e., tracheostomy, post-surgical edema). Increased operating time [11, 16–18], multiple surgeries/revision surgeries [16, 18–20], cervical surgeries, and anterior cervical discectomy and fusion (ACDF) surgeries [14, 16, 20–24] are all associated with increased risk of dysphagia.

There is growing literature on the incidence and risk factors of dysphagia in patients with cSCI (Table 1). However, there are several limitations in the existing literature. Most studies only evaluated patients who were admitted to specialist spinal cord injury units, which may exclude patients with co-morbidities or cognitive impairments who would be managed in different settings such as traumatic brain injury units. Secondly, some studies focus on patients with elective spinal surgeries, traumatic injuries, or a mixture of both types of surgeries. These differences in patient etiology *and* clinical management have implications on the risk factors for dysphagia and expected pathophysiology. Lastly, the diagnosis of dysphagia varies depending on the method of evaluation (i.e., bedside swallowing evaluation versus instrumental assessment) and expertise of the assessor [i.e., nurse versus speech–language pathologist (SLP)]. Patients with cSCI are at increased risk of silent aspiration due to paralysis of the respiratory muscles and/or blunted laryngeal and tracheal sensation [25], further limiting the diagnostic accuracy of a bedside swallowing screen for dysphagia diagnosis.

**Table 1:** Summary table of studies reporting dysphagia following cSCI

Authors	Study site, period of assessment, inclusion	Study size (*n*)	Etiology	Mean age (range); M:F	Screen and assessment tools	Dysphagia incidence (%)	Correlating factors	Recommendations
Kirshblum et al.1999 (R) [11]	on admission to rehabilitation unit; Acute traumatic SCI	187	Trauma	44.3 (15–86) 5:1	BSE, MBT, VFSS	22.50%	Age, tracheostomy, ventilation, anterior cervical surgery	Early diagnosis
Wolf & Meiners 2003 (P) [26]	within 3 months of admission to spinal unit; Acute cervical lesion	51	Trauma 46 Non-trauma 5	43.4 (16–89) 2.2:1	FEES	80%	Brainstem lesions, NOT age or level, anterior surgery	Early treatment
Brady et al. 2004 (R) [27]	on admission to two rehabilitation units; All cervical injuries	131	Trauma and non-trauma	55.6 (17–87) 1:1.2	BSE, VFSS/FEES	55%	Tracheostomy, cervical spinal surgery, brain injury	Identify dysphagia using predictive factors
Abel et al.2004 (P) [28]	on admission to spinal unit; cSCI	73	Trauma 56 Non-trauma 17	42.9(0.57–86.8) 2.3:1	Questionnaire, MBT, VFSS	44%	High cervical and complete injuries, tracheostomy	Early detection and monitoring
Seidl et al. 2010 (R) [29]	Within 8 weeks of admission to trauma center; C0-C8	175	Trauma 147 Non-trauma 28	43.45 (14–89) 4.6:1	BSE + FNE	16%	Level of paralysis, tracheostomy, ventilation, other injuries	SLP assessment pre-oral feeding, FNE if dysphagia is suspected
Shin et al. 2011 (R) [10]	Inpatients admitted to spinal unit; All tetraplegic patients	121	Trauma 118 Non-trauma 3	44.93 (9–78) 6.6:1	VFSS	8%	Age, tracheostomy, dysphagia signs	Monitor for signs of aspiration
Shem et al. 2011 (P) [30]	Acute cSCI within 31 days of injury	29	Trauma	41 3.1:1	BSE and VFSS	41%	Age, tracheostomy NG tube	Early screening
Chaw et al. 2012 (P) [31]	Within 32 days of admission to spinal unit; Acute cSCI	68	Trauma and non-trauma	43 (range not given) 5:1	BSE and VFSS within 72 h	30.90%	Ventilation, tracheostomy, NG, age	Need good pulmonary management
Shem et al. 2012 (P) [32]	All admissions to spinal unit; Acute tetraplegia	40	Trauma	41 (23.5–68.7) 3.4:1	BSE and VFSS	40% based on BSE; 44% on VFSS, 14.8% with aspiration	Age, tracheostomy, ventilation, and NG tube	Early screening of all tetraplegic patients
Lee et al. 2016 (R) [22]	All cSCI admissions to trauma center	56	Trauma	Not available	Bedside nurse screen and SLP assessment (decannulated)	41% (56 patients has cSCI of which 23 had dysphagia)	Age, spinal cord injury	Elderly and cervical injury should be monitored for risk of dysphagia
Hayashi et al. 2017 (R) [14]	Traumatic cSCI admission to spinal injuries center within 3 days	298	Trauma	64 (14–91) 6.1:1	Based on tube dependence due to aspiration	7.0%	Age, severe paralysis, tracheostomy	Evaluate risk factors to identify dysphagia
Ihalainen et al. 2017 (P) [33]	Acute cSCI admitted to hospital	46	Trauma	62.1 5.5:1	VFSS	41% penetrated 33% aspirated of which 73% silent aspiration		VFSS recommended Swallow evaluated by speech and language therapist
Ihalainen et al. 2018 (P) [34]	cSCI admitted to hospital	37	Trauma	61.2 5.2:1	Clinical swallowing trial and VFSS on all patients at 28 days	51.4% penetrators-aspirators; 71.4% silent aspiration	Need for bronchoscopy, lower level ACSS, coughing, throat clearing, choking, voice quality changes	Use risk factors to initiate preventative measures
Shem et al. 2019 (P) [15]	Adult patients admitted to SCI inpatient rehabilitation unit	76	Trauma	48 ± 19	BSE and VFSS	30% based on BSE; VFSS (n = 17) 0f which 82% dysphagia, aspiration 21.4%	Tracheostomy, invasive mechanical ventilation, nasogastric tube, history of pneumonia, and older age	Early screening in acute cSCI
Hayashi et al. 2020 (P) [35]	Traumatic cSCI admission to spinal injuries center within 2 weeks of injury	136	Trauma	65.1 ± 14.1 years	Dysphagia Severity Scale, width of retropharyngeal space	32%	Age, ASIA motor score, tracheostomy, and swelling of retropharyngeal space	Morphological changes to pharynx affect dysphagia
Hayashi et al. 2020 (P) [36]	Traumatic cSCI admission to spinal injuries center within 2 weeks of injury	65	Trauma	67 (60–73 IQR) 14:51	Dysphagia severity scale (DSS) and functional oral intake scale (FOIS), supported by FEES and VFSS	35% reducing to 17% at 3 months	Severity of motor score	Monitor CSCI patients in 2 weeks after injury and those with low motor scores

*R* retrospective, *P* prospective, *MBT* modified blue-dye test, *VFSSS* videofluoroscopic swallow study, *FEES* flexible endoscopic evaluation of swallowing, *BSE* bedside swallow evaluation, *FNE* flexible nasendoscopic evaluation

## Causes of Dysphagia in cSCI

In the following section, we discuss the possible causes of dysphagia following cSCI.

### Upper Spinal Cord Anatomy/Neurology

Temporary or permanent nerve damage related to the injury itself can directly contribute to dysphagia pathogenesis. Lower cranial nerves that play a key role in swallowing, glossopharyngeal (IX), vagus (X), and hypoglossal nerves (XII), are vulnerable to damage from cSCI due to compression of the brainstem [37]. Cervical spinal nerves also contribute to safe and efficient swallowing. The ansa cervicalis is a combination of fibers that include the hypoglossal and cervical spinal nerves (C1–C3)[38]. It provides motor innervation to the geniohyoid muscle as well as the strap muscles (omohyoid, sternohyoid, sternothyroid, thyrohyoid), which contribute to hyolaryngeal excursion and airway protection during swallowing [38]. In addition to neural impairments, anatomical alterations in the upper airway post injury can impair swallowing function. Patients with cervical kyphosis (i.e., abnormal curvature of the cervical spine) have been shown to have increased hypopharyngeal transit times and impaired airway protection [39]. The impact of atypical anatomy on swallowing function in patients with cSCI, who often have additional spinal fusion hardware and soft tissue thickening/edema, is not well documented but this is likely a contributor to dysphagia in this population [35]*.*

### Spinal Surgery Consequences

**Fig. 1:**
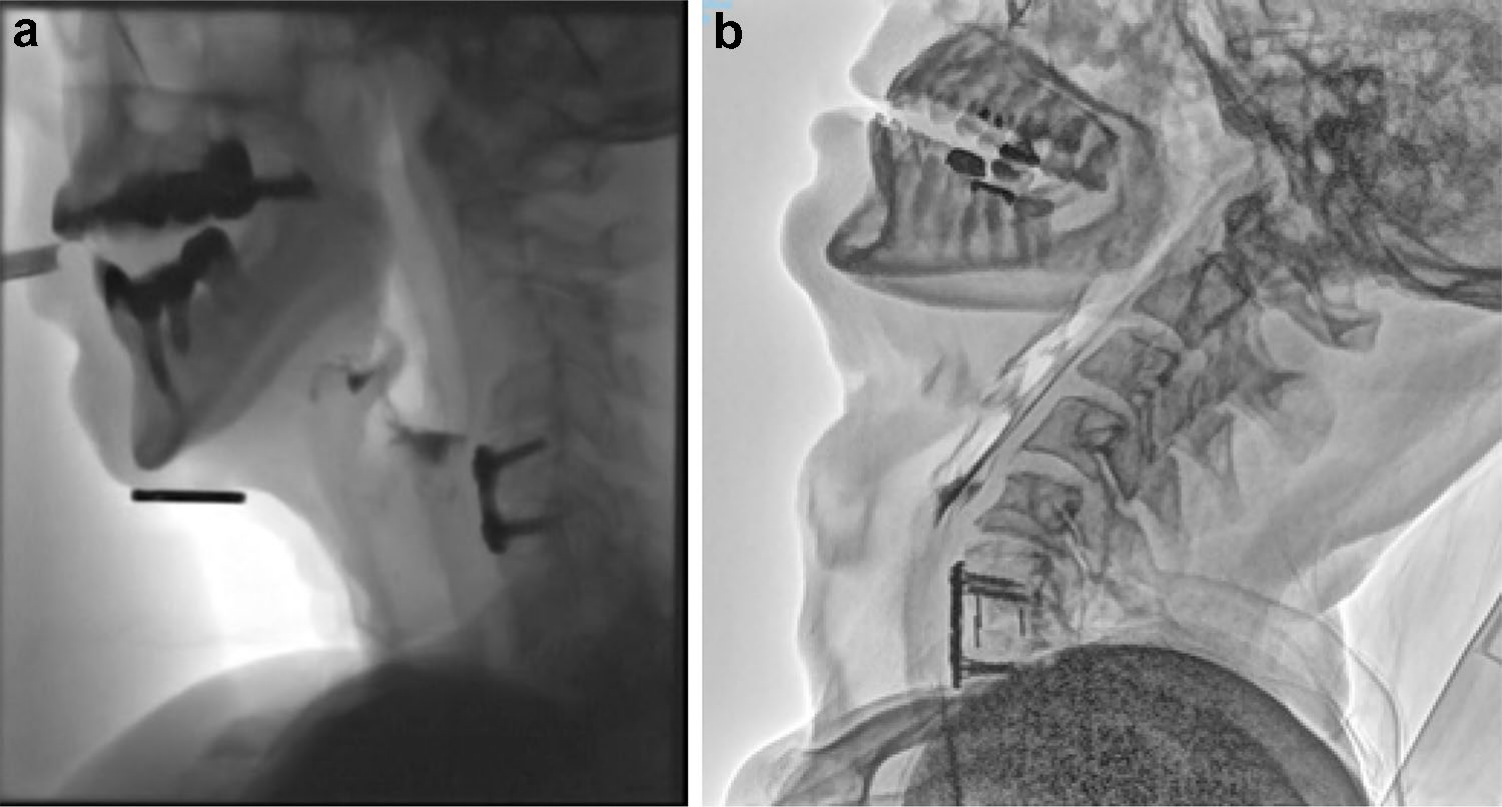
**a** Radiographic image of a C4-5 plate with posterior pharyngeal wall thickening (3 weeks post surgery). **b** Radiographic image of C6/7 anterior fixation with plate with altered C-spine alignment (5 months post surgery) (with permissions)

The potential impact of operative approaches and techniques have been well described in a number of review papers over the last 10 years, including detailed recommendations to reduce the risk of postoperative dysphagia [16, 18]. The dC2–C7 angle is thought to play an important role in the development of dysphagia in both anterior and posterior cervical spine surgery [23]. Intra-operative measurement of the dC2–C7 angle is practical and essential in avoiding inadvertent postoperative swallowing difficulties. Complications related to cSCI surgery, such as compression in the cervical spine related to hardware placement, altered pharyngeal and spinal structure, and sensory impairments related to temporary or permanent neuropathy and/or edema are known risk factors for dysphagia (Figs. 1a and b). The prominence of the plate can impact on bolus flow and thicker plates have been associated with greater risk of dysphagia [40]. Halo braces and neck collars have been shown to affect swallowing biomechanics in terms of timing and displacement in healthy adults [41, 42] and therefore may add to swallowing difficulties a patient may be experiencing.

### Respiratory Muscle Dysfunction and Dysphagia

Paralysis or impairment of the major respiratory muscles with injuries above C5 interrupts normal breathing patterns, which are essential for safe and efficient swallowing [43, 44]. Precise respiratory–swallowing coordination is vital for airway protection [43]. Expiration before and after swallowing is the most common breathing pattern in healthy adults [44, 45]. It serves to expel any misdirected materials from the airway after swallowing, offering an additional airway protective mechanism. Deviation from this respiratory–swallowing pattern is associated with increased risk of airway invasion [43]. Initiation or completion of swallowing with an inspiration serves to bring air and potentially misdirected food or fluid particles into the lungs [43, 46]. There are no studies that specifically evaluate respiratory–swallowing coordination in patients with cSCI. However, patients with cSCI have reduced lung volumes and shortness of breath [47]. This atypical breathing pattern is likely to contribute to respiratory–swallowing discoordination and increased risk of airway invasion.

The phrenic nerve (C3-C5) provides exclusive motor control to the diaphragm, the primary muscle for inspiration. There is strong evidence for the role of the diaphragm in safe swallowing [48]. Active contraction of the diaphragm creates an active breath hold or ‘swallow breath’ during swallowing [48, 49]. This preserves respiratory volume for post-swallow expiration that is important for airway protection and airway clearance [48]. Activation of the diaphragm creates negative trans-diaphragmatic pressure [50, 51]. During swallowing, this contributes to a favorable pressure gradient (i.e., a suction effect) for bolus flow across the upper esophageal sphincter [51]. Recent studies in animal models of cSCI demonstrate that the absence of diaphragmatic activation during swallowing disrupts negative pressure generation in the esophagus and results in disordered laryngeal muscle activity, contributing to penetration and aspiration [52]. These preliminary findings suggest that damage to the phrenic nerve and diaphragm innervation following cSCI may also directly contribute to swallowing dysfunction.

Respiratory muscle dysfunction associated with cSCI can also impair airway clearance mechanisms that are vital for safe ingestion of food and fluid. The cough response protects the airway from foreign bodies or irritants, including mis-directed food, fluid, and saliva. The intercostal and abdominal muscles are important for coughing and effective airway clearance. Following cSCI, cough is impaired to various degrees depending on the level and completeness of the injury with more dysfunction observed at higher levels of injury [25]. In patients with complete cSCI at C5 and above, all measures of respiratory function (including forced vital capacity, forced expiratory volume, and peak expiratory flow rate) were found reduced by at least 50% of predicted normal values [53]. This indicates that the cough response to aspiration of food, fluid, and saliva is likely impaired in these patients. Additionally, reduced respiratory and cough function are associated with increased risk of penetration, aspiration, and aspiration pneumonia across a range of patient populations [54–58]. This is due to the shared anatomical and neural substrates of coughing and swallowing. Furthermore, laryngeal muscle impairments due to direct injury, or management of the spinal injury, can result in vocal fold dysfunction and poor glottal adduction resulting in reduced cough efficiency and effectiveness [59].

### Esophageal Impairment and Oro-Pharyngeal Dysphagia Following cSCI

While the focus of this review is on oro-pharyngeal dysphagia in cSCI, the relationship between oropharyngeal and esophageal impairments is well documented in the literature [60–62]. Research suggests that esophageal symptoms are often under-reported in patients with SCI [63], therefore, dysphagia clinicians should take an active role in screening and referring patients to gastroenterology, given the potential for adverse consequences on swallowing safety and efficiency. In addition, given the functional interrelationship between the pharynx and esophagus, pharyngeal impairments may be impacted by esophageal abnormalities [61]. Patients with cSCI are at high risk of esophageal impairments [60, 64]. Autonomic dysfunction following cSCI can cause dysmotility of the gastrointestinal system leading to paralytic ileus [65]. Second, damage to upper esophageal mucosa can occur from penetrating traumatic injuries, such as knife stabbings and gunshot wounds and as a complication of cervical spinal surgery, due to displaced screws or metal work [66, 67]. These esophageal changes can promote esophageal dysmotility, gastroesophageal reflux disease (GERD), and upper esophageal dysfunction [60, 68].

### Dysphagia due to Medical Management

For traumatic cSCI patients, the early focus is on maintaining good respiratory function and minimizing neurological damage following injury to the spine. For injuries C5 and above, there is a high risk of respiratory failure due to paralysis of respiratory muscles that will necessitate the need for mechanical ventilation [47, 69]. Although non-invasive ventilation (NIV) and negative pressure options are available [68], usual practice is to insert an alternative airway, in the form of a tracheostomy to assist with both ventilation and toileting/secretion management. Both tracheal intubation and tracheostomy can cause a range of acute and chronic laryngeal complications that contribute to dysphagia [70, 71]. Potential complications include laryngeal edema, vocal cord palsy, sensory impairments, stenosis, and granuloma and readers are directed to Wallace and colleagues for a thorough review of this evidence [70].

Numerous studies have demonstrated that the presence of a tracheostomy is a significant independent risk factor for dysphagia after cSCI [14, 31, 72–74]. One study reported a threefold risk (RR: 3.67) of dysphagia with a tracheostomy following cSCI [73]. The cause of dysphagia following tracheostomy is complex and multifactorial. The presence of a tracheostomy substantially alters pharyngeal and laryngeal biomechanics. It impairs hyolaryngeal excursion that is necessary for airway protection during swallowing and opening of the upper esophageal sphincter for bolus transport to the stomach [10, 11, 14]. Changes in subglottic air pressure (with an open versus close tracheostomy tube), are known to affect pharyngeal swallowing physiology, including bolus transit times and airway invasion [75]. The absence of subglottic air pressure during swallowing may disturb the favorable pressure gradient for movement of the bolus into the esophagus [51]. The presence of a tracheostomy and need for ventilation creates an alteration to respiratory–swallowing coordination with increased risk of aspiration [76]. Further desensitization of the upper airway due to tracheostomy may affect airway protection and cough sensitivity in acute patients. Disruption to vital capacity leads to respiratory muscle fatigue and a failure to wean from ventilator support [77].

## Clinical Presentation and Assessment of Dysphagia in cSCI

Table 2 provides a summary of published data describing the features of dysphagia in cSCI from instrumental assessment findings. In the acute phase, aspiration, pharyngeal residue, decreased or absent hyolaryngeal elevation, impairments in hyoid displacement, pharyngeal constriction, abnormal pharyngeal wall thickness, reduced pharyngoesophageal segment opening, reduced epiglottic deflection, and reduced pharyngeal constriction are commonly observed [78–83]. These symptoms, in the majority of cases, are expected to resolve 2–6 months post surgery [78], suggesting that patients are most vulnerable to dysphagia, aspiration, and aspiration pneumonia in the acute phase post injury.

**Table 2:** Summary table of studies identifying dysphagia characteristics in cSCI using instrumental tools

Paper	Population	Tool used	Dysphagia characteristics
Bekelis et al. 2010 Case report [79]	61-year-old male Traumatic cSCI C1-C3 fusion (posterior approach)	FEES and VFSSS	Bilateral vocal cord paresis; at 1 month reduced epiglottic inversion, reduced hyolaryngeal elevation, and hypokinesis of pharyngeal wall Required PEG and returned to modified diet
Cumpston and Bock 2015 Case report [80]	84-year-old male Traumatic SCI C1-2 fusion (posterior approach), projection of screw seen at C1 into retropharynx	VFSSS	↓ pharyngeal constriction & laryngeal elevation Minimal tongue base retraction Required PEG & resolved spontaneously
Dettling et al. 2013 Case report [81]	16-year-old male Traumatic SCI—halo fixation	FEES & VFSSS	↓ soft palate movement, pooling secretions, aspiration Required NGT & resolved spontaneously
Dick et al. 2020 Experimental case series [82]	4 patients Two traumatic and two non-traumatic cervical spine injuries	VFSSS (quantitative measures)	↓ anterior hyoid excursion, ↓ pharyngeal constriction, ↓ UES opening, ↑ pharyngeal wall thickness Three returned to oral diet, one remained NBM
Hamilton et al. 2022 Prospective observational [83]	20 traumatic cSCI patients	VFSSS	↓ pharyngeal constriction, ↑ time to reach peak hyoid excursion, delayed and incomplete laryngeal vestibule closure
Miles et al. 2021 Retrospective observational [78]	62 patients (traumatic & non-traumatic (85% cervical spinal injuries)	62 FEES 11 VFSS	↓ pharyngeal constriction &↓ hyoid displacement, ↓ UES opening with residue, aspiration & secretion accumulation

*VFSS* videofluoroscopic swallow study, *FEES* flexible endoscopic evaluation of swallowing, *PEG* percutaneous endoscopic gastrostomy, *NGT* nasogastric tube, *UES* upper esophageal sphincter, *NBM* nil by mouth

### Screening and Assessment of Dysphagia in cSCI

Early detection of dysphagia is therefore of high clinical value to prevent aspiration pneumonia. However, despite the known risks of dysphagia following cSCI, no routine method of screening exists [11]. One of the key challenges in this patient population is that patients with cSCI with tracheostomy may not demonstrate overt signs of laryngeal penetration or aspiration (e.g., coughing after food/liquid intake) due to reduced or absent laryngeal sensation and/or weak cough—a phenomenon known as silent aspiration. This makes it challenging for healthcare staff to screen for dysphagia prior to referral to SLP for instrumental swallowing assessment. Clinical or bedside swallow screening requires administration of food or fluid trials to evoke adverse motor response such as coughing to determine a patient’s swallowing dysfunction.

Validated dysphagia screening tools have been evaluated with patients with cSCI. Posillico, Golob [84] used the Yale Swallow Protocol [85] with all patients admitted with a suspected spinal cord injury. The authors reported a 16.7% incidence of dysphagia. This bedside screening tool had high sensitivity (84.2%) and specificity (95.8%) for detecting dysphagia. However, 48.4% of the 221 patients were excluded from the screening process because they were critically ill and instrumental assessments were only conducted on those who failed their Yale. Thus, the sensitivity and specificity of the tool for detecting dysphagia in a true clinical cohort of cSCI patients is unclear. Other dysphagia screening tools with high sensitivity and specificity have been developed for stroke and neurogenic populations. These include the water swallowing test, the Mann assessment of swallowing ability (MASA) [86], and the Gugging swallowing screen (GUSS) [87]. However, their sensitivity and specificity for identifying dysphagia and aspiration in patients with cSCI are currently unknown and may, again, be limited given the unique pathogenesis and risk factors of dysphagia in cSCI. In response to this, The Dysphagia following Acute Cervical Spinal Cord Injury (DAISY) swallow screening tool [88] was developed through international expert consensus. It focuses on identifying risk factors for dysphagia and presence of clinical symptoms suggestive of aspiration in cSCI, i.e., injury risk, clinical risk, and urgency (Table 3). This tool enables healthcare staff to identify risk factors for dysphagia prior to commencing oral trials and preventing adverse outcomes. If risks are evident a referral to SLP or another dysphagia clinician is recommended for diagnostic instrumental assessment. If clinical symptoms of dysphagia are already evident then the team are instructed to review and change current clinical management and identify possible causes. This may highlight processes such as mouth care or managing thirst that may be exacerbating dysphagia. Further validation of this screening tool is planned alongside a SCI-specific FEES protocol.

**Table 3:** Domains, category, and sub-category of DAISY swallow screening tool

Domains	Category	Sub-category
Injury risk	Comorbid	Brain injury/cognitive deficit
	Level of injury	Cervical SCI C1-C7
	Severity of injury	Complete/incomplete injury
	C-spine surgery	Anterior or posterior cervical spine surgery
Clinical risk	Intubation	> 48 h
	Tracheostomy	Cuffed or uncuffed tube
	Ventilation	Requiring up to 24-h ventilation
	Nutrition	Reduced nutritional intake
Urgency	Chest infection	Recent chest infection
	Pyrexia	Spiking pyrexia
	Oral hygiene	Increased need for oral care
	Suction	Increased need for suction

Cough reflex testing is a screening tool that is used in other patient populations with neurogenic dysphagia (e.g., acute stroke, Parkinson’s Disease) to identify risk of silent aspiration. It involves inhalation of a tussive (i.e., cough evoking) agent, most often citric acid or capsaicin, at concentrations that are known to elicit a cough response in healthy individuals. In the clinical setting, cough reflex testing is used in the acute stages to support clinical decision-making regarding patients’ risk of silent aspiration [89]. To date, no studies have evaluated the validity of cough reflex testing in patients with acute cSCI and dysphagia for this purpose. Lin and colleagues [90] evaluated citric acid cough thresholds (i.e., the concentrations of citric acid that were required to elicit a cough response) in healthy individuals and individuals with chronic thoracic and cSCI (1–20 years post injury). They found that cough thresholds were reduced in those with chronic SCI, compared to the control group, meaning that individuals with chronic SCI had *enhanced* upper airway sensation i.e., hypersensitivity [91]. In contrast, Dicpinigaitis and colleagues [25] found no difference in capsaicin cough threshold in individuals with chronic cSCI (5–41-year post injury) and healthy controls. While capsaicin is known to target different cough mechanisms to citric acid [92], the findings suggest that upper airway sensation may be intact, or in fact enhanced, in chronic SCI. However, it is highly likely that blunted laryngeal sensation contributes to aspiration and silent aspiration in the acute phase post cSCI, especially in patients who are intubated [93]. Studies are needed to determine the validity of cough reflex testing for identifying silent aspiration in acute cSCI.

### Instrumental Assessment

Swallow screening establishes those likely to have dysphagia and clinical swallowing evaluations at bedside may enable clinicians to hypothesize about possible swallowing difficulties and gauge a patient’s risk of aspiration and aspiration pneumonia. However, instrumental assessments, namely, Videofluoroscopic Swallow Study (VFSS), Flexible Endoscopic Evaluation of Swallowing (FEES), and High-resolution Impedance Manometry (HRIM) allow clinicians to evaluate swallowing biomechanics to inform rehabilitation and management. VFSS has been reported more frequently in research studies with cSCI patients. However, it is important to note that with VFSS and HRIM, there is a requirement for the patient to be able to tolerate transport to radiology or gastroenterology clinics and maintain an upright seating position. Thus, findings from VFSS and HRIM studies may be bias towards people with less severe or acute cSCI.

FEES is a more accessible tool in the acute and critical care settings as it can be performed at a patient’s bedside. FEES provides detailed imaging of the pharyngeal and laryngeal structures; however, it does not provide a view of oral stage activity. FEES enables evaluation of secretions and airway patency, facilitating clinical decision making for a patient’s ability to tolerate oral trials. Clinical settings can vary in their access to these instrumental assessments and the availability of SLP services, affecting dysphagia diagnosis and development of swallow rehabilitation programs [94].

## Treatment Approaches for Dysphagia After SCI

### Clinical Management of Dysphagia in cSCI

Working as a multidisciplinary team can maximize outcomes for patients with a multifaceted approach [95, 96]. Patients with cSCI need a personalized care approach to tracheostomy and ventilator weaning that takes into consideration respiratory muscle paralysis and fatigue that reduces the usual pace of weaning [97, 98]. SLPs have a role to play in evaluating laryngeal function alongside the weaning process [70]*.* There is limited published research in SLP rehabilitation for patients with cSCI. This is perhaps in part due to the heterogeneous population and the challenges of doing research in patients with acute illness. As a result, SLPs need to utilize advanced clinical reasoning and evidence from other patient populations in managing dysphagia in patients with cSCI. Despite the paucity of evidence, it is reasonable to consider that optimizing certain factors may increase the likelihood of improved dysphagia outcomes. These include positioning, management of patients’ psychological wellbeing, and management of dry mouth and taste and smell changes. In addition, given the level of dependency, those supporting patients with oral care and at mealtimes should have the required training to do so. The need for SLP intervention is recognized [1, 21] and some of the areas of treatment currently in practice are summarized in Table 4, alongside emerging therapies.

**Table 4:** Multidisciplinary treatment approaches for patients after SCI

Timing	Intervention	Literature/Evidence
Early interventions	Secretion management (hypersalivation vs. dry mouth)	[99–101]
	Tracheostomy manipulation for swallow therapy	[102]
Rehabilitation	Treating the neurological impairments—swallow exercises	[82, 103]
	Ear Nose Throat surgeries, e.g., vocal fold augmentation	[104–106]
	Respiratory therapies incl. EMST	[31, 107, 108]
	Cough therapies	[109]
	Emerging Therapies	
	Respiratory Muscle Training	[110]
	Abdominal functional electrical stimulation	[111]
	Acute Intermittent Hypoxia	[112]

### Swallowing and Respiratory Muscle Training

Dick et al. [82] recently published a case series documenting the effects of an individualized swallowing intervention for four patients cSCI (2 traumatic, 6 weeks-10yrs since injury). Intervention was tailored to participants underlying physiologic impairment (i.e., reduced pharyngeal constriction, impaired hyoid displacement, etc.) identified on instrumental assessment, and involved exercises such as the effortful swallow, Mendelsohn maneuver and chin tuck against resistance. Improvements in timing and swallowing biomechanics were observed post intervention. Functional swallowing improvements were also noted, with three out of four patients able to have their feeding tubes removed post intervention. This study highlights the benefit of tailored interventions to improve swallowing outcomes in patients with cSCI.

A recent systematic review by Berlowitz and colleagues [110] evaluated the effects of respiratory muscle training in patients with cSCI. Respiratory muscle training comprised specific training of inspiratory and/or expiratory muscles using a device or singing in one study. Results indicated that respiratory muscle training was safe in patients with cSCI and no adverse effects were noted. Increases in vital capacity, maximum inspiratory pressures, and maximum expiratory pressure were reported post intervention. While the precise effects on swallowing outcomes remain unclear, the findings suggest that respiratory muscle training could enhance cough, swallowing, and airway clearance mechanisms for patients with cSCI based on previously published literature documenting the relationship between improvements in aerodynamic measures and swallowing safety in other neurogenic populations [55, 56, 113, 114]. This is further supported by research demonstrating positive effects of expiratory muscle strength training (EMST) on swallowing safety and airway protective mechanisms in patients with neurogenic dysphagia [115–119]. Furthermore, enhancing respiratory muscle strength is known to decrease risk of aspiration pneumonia [58, 120, 121]. Studies to evaluate the effects of respiratory muscle training on swallowing outcomes in patients with cSCI are urgently needed.

### Abdominal Functional Electrical Stimulation

Abdominal functional electrical stimulation (FES) involves the application of surface electrodes that emit electrical pulses (20–50 Hz) to the abdominal muscles to achieve muscle contraction. Recent studies demonstrated that abdominal FES could improve forced vital capacity, vital capacity, peak expiratory flow, and unassisted cough production in patients with tetraplegia [111, 122, 123]. To date, no study has evaluated swallowing specific outcomes following abdominal FES in patients with cSCI. However, the findings suggest that abdominal FES could enhance airway clearance mechanisms and swallowing outcomes [55, 56, 113, 124]. Including swallowing outcomes in future clinical trials on FES is required.

### Acute Intermittent Hypoxia (AIH)

There is growing interest in the use of therapeutic AIH to enhance limb and respiratory function in patients with cSCI [125]. AIH involves exposing individuals to bouts of low oxygen interspersed with normal oxygen levels to enhance plasticity in spinal synaptic pathways. It can be combined with other treatments such as drug treatments, cortical or spinal stimulation, and task-specific training to optimize functional outcomes [125]. Studies have shown that AIH enhances limb and respiratory function in animal models [126, 127] and patients with cSCI [128–130]. The greatest functional effects are observed when AIH precedes task-specific motor training, in that, AIH serves as a ‘plasticity primer’ [112]. The precise underlying mechanisms of this therapeutic effect are still under investigation. However, results suggest that AIH combined with swallowing-specific training may enhance swallowing function for patients with cSCI. AIH interventions are highly novel and remain in Phase I/II clinical trials and are not yet ready for widespread clinical implementation.

### Barriers to Rehabilitation

There are many unique challenges to rehabilitation in this population that we do not encounter in neurorehabilitation of other patients populations. Bracing, collars, and neck fixation alters and restricts neck position during mealtimes often with a tendency for a slightly superiorly tilted head posture rather than a perhaps more protective chin tucked position [41, 131]. Bracing, collars, and neck fixation also make some compensatory swallowing strategies such as head tilt, turn, and chin tuck maneuvers difficult or prohibitive and may make some rehabilitation exercises unmanageable such as the Shaker head lift [82]. Psychological impacts of injury including fear, anxiety, and low mood may all make return to oral intake as well as exercise adherence a challenge [132, 133]. Some services are heavily focused on independence of mobility and self-care for rehabilitation discharge with little focus on swallowing and voice recovery [134]. Living with a spinal injury comes with many other complications that can impact on rehabilitation including hypotension, risk of pressure areas, and trunk and head control. Pain and fatigue during movement may also reduce rehabilitation capabilities. Appetite, taste, and smell disturbances impact motivation to return to oral intake.

### Implications for Clinical Practice

While initial treatment of a patient with a SCI will take place in their acute center, ongoing rehabilitation will take place in either a specialist SCI center or non-specialist rehabilitation center. Specialist centers are established in a number of high-income countries, however, in these countries, there may be a limited number of specialist beds available. A recent report in the UK identified reduced bed capacity in specialist units resulting in cSCI patients remaining in non-specialist units for prolonged periods of time [135]. For those patients requiring ventilator support, bed capacity is even more restricted with many units taking small numbers due to the high care demands and staffing requirements.

It has been suggested that early admission to a specialist spinal injury center can improve patient outcomes and that dysphagia management practices differ between specialist and non-specialist settings. A survey conducted by McRae et al. [136] aimed to explore clinical practices within specialist and non-specialist critical care units in the management of cSCI patients with respiratory and swallowing impairments. This study identified an increased use of instrumental swallow studies in specialist centers and increased use of cuff deflation during weaning to help restore verbal communication and support swallowing. These differences would suggest that a patient’s dysphagia rehabilitation is optimized in a specialist center with access to a specialist dysphagia team including a Speech–Language Pathologist (SLP). There is an indication that those receiving rehabilitation in a specialist center achieve greater functional gains than those who do not [137]. This is supported by McRae’s findings [136] that non-specialist centers had lower expectations for patients returning to safe eating and drinking and an increased reliance on diet and fluid modifications to manage dysphagia. As part of the patient pathway, they will often transition from non-specialist to specialist centers. This difference in dysphagia management can leave patients feeling confused and disengaged with the rehabilitation process. The need for a consistent multidisciplinary team (MDT) approach to dysphagia management is therefore required. Many SCI centers have outreach teams, and providing they include a SLP, this may go some way to bridging this skills and knowledge gap and ensuring a consistent approach to dysphagia management.

Length of stay in inpatient rehabilitation units can vary considerably and the factors that contribute to this are myriad. Those with a higher, more severe spinal cord injury have a longer length of stay than those with incomplete paraplegia. Associated complications including pressure sores, poor nutritional status, and respiratory compromise can also increase length of stay. The average length of stay for a cSCI in the UK is 3 months, in contrast the median length of rehabilitation stay in the US is 32 days [138]. To varying degrees depending on the country and setting, a patient’s discharge date will be determined by their medical stability, functional gains, motivation for therapy, funding, and access to a suitable discharge destination. In U.K. health systems, a goal-focused approach is utilized, whereby inpatient stays will be extended if appropriate patient goals are identified. Once a patient is discharged, there are logistical and service provision barriers to accessing an instrumental swallow assessment and intensive dysphagia therapy. Given these pressures, specialist, targeted input from appropriately trained clinicians is vital to optimize patient outcomes during inpatient stays.

Best practice guidelines should be used to ensure patients access specialist care at the right time and ensure a proactive approach to identifying dysphagia based on clinical risk factors is employed. Access to a SLP with specialist skills in cSCI can help improve dysphagia management. Further research into optimal models of care for cSCI patients is needed to inform service development worldwide, this should include longitudinal data examining long-term outcomes following admission to specialist and non-specialist centers.

## SLP Service Provision

There is increasing evidence that recognizes the need for SLPs to be clinically involved in the management of swallowing and communication impairments in people with cSCI during acute care, rehabilitation, and following discharge into the community [1]. Despite this there are currently no recommendations for levels of SLP staffing required for an optimal service to those with cSCI. A recent service evaluation in UK has highlighted that services in spinal units have limited staffing provision compared to those in major trauma centers, which reduces the range and intensity of therapy [94]. In the UK, a collaboration of professional bodies have proposed a minimum standard for SLP service provision to SCI rehabilitation to be delivered as part of a team approach [139] (Table 5).

**Table 5:** Minimum standards of Speech–Language Pathology service delivery to SCI rehabilitation [139]

1. Access to Speech–Language Pathology for a minimum of five days a week for patients with communication and swallowing difficulties on admission is mandatory and should be part of the multidisciplinary team assessment
2. A baseline assessment must include case history, cranial nerve assessment, secretion management, voice, communication (both speech and language), cognition, oral health, swallowing, associated respiratory function and outcome measures
3. The therapist may be required to undertake instrumental assessment, as appropriate, including videofluoroscopy, Fibreoptic Endoscopic Evaluation of Swallow (FEES), Ultrasound, manometry and surface EMG for the assessment of laryngeal function and airway patency for management of weaning, dysphagia and communication difficulties
4. The rehabilitation process should include: a. targeted and physiologically specific therapeutic interventions b. biofeedback tools to enhance patient involvement, e.g., surface EMG, FEES, respiratory muscle strength training c. opportunities for oral trials d. optimizing secretion management and oral care e. improving breath support for phonation f. access to alternative and augmentative communication g. utilizing above cuff vocalization
5. There should be access to instrumental assessment of swallow, either FEES (during acute phase when tracheostomy is placed and patient is extubated) and VF for returning to oral intake or to support tracheostomy and ventilator weaning
6. The assessment of voice and breath support for adequate communication is essential
7. Other areas will include rehabilitation of swallow, communication and advice on mouth care

## Conclusions

The identification and management of dysphagia in cSCI is an emerging area of clinical practice and dysphagia clinicians have a key role to play. Dysphagia clinicians need specialist understanding of the mechanisms of dysfunction and suitability of interventions. This group of patients will require lifelong care with a multi-professional focus to deliver interventions dependent on changing needs. Swallowing and communication impairments may be neglected due to other healthcare issues but these remain a priority for those living with a cSCI and contributes to their quality of life. A dysphagia team with a SLP should be considered a part of core services with dedicated staff.

## Supplementary Information

Below is the link to the electronic supplementary material.

Supplementary file1 (DOCX 12 KB)
