# Supplementary material for: Oropharyngeal Dysphagia in Acute Cervical Spinal Cord Injury: A Literature Review
Source: Dysphagia. 2022 Nov 14;38(4):1025–38. doi: 10.1007/s00455-022-10535-0 (PMC10326135; doi:10.1007/s00455-022-10535-0)
Supplement: Supplementary file 1 — Supplementary file1 (DOCX 12 KB) [file 455_2022_10535_MOESM1_ESM.docx]

**Search terms for literature search:**

**Databases searched:** Medline and Pubmed

**Limits applied:** Human, English language

**Publications in last 20 years**

**Search terms used:**

Oropharyngeal dysphagia, Spinal cord injury, spinal surgery injury, Deglutition, Deglutition disorders, Swallow, Dysphagia, Cervical spinal cord injury, Cervical spinal injury, Spinal cord injury, Spinal injury, Spinal cord injuries

82 articles found

Excluded:

Spinal surgery only, surveys, systematic reviews, noninterventional studies

Included only those reporting incidences of dysphagia in acute cSCI where instrumental and or bedside swallowing assessment was used.

**16 articles included**
